# Knot formation in a thoracic epidural catheter: a case report

**DOI:** 10.1186/s40981-021-00448-6

**Published:** 2021-06-07

**Authors:** Toshiyuki Mizota, Kayo Kimura, Chikashi Takeda

**Affiliations:** Department of Anesthesia, Kyoto University Hospital, 54 Shogoin-Kawahara-Cho, Sakyo-Ku, Kyoto, 606-8507 Japan

**Keywords:** Epidural anesthesia, Knot, Thoracic

## Abstract

**Background:**

Although most epidural catheter knot formation has been reported in lumbar epidural catheter placement, knot formation in a thoracic epidural catheter has been experienced.

**Case presentation:**

A 72-year-old woman was scheduled for laparoscopic cholecystectomy under general anesthesia combined with epidural anesthesia. The epidural catheter was inserted through the Th10–Th11 intervertebral space and was placed 7 cm into the epidural space. Two days after the surgery, the anesthesiologist was called because of difficulty in removing the epidural catheter. The catheter was eventually removed when the anesthesiologist carefully pulled it while strongly bending the patient’s body to the right, although resistance was still noted. The removed catheter was observed to have a hard single knot formed at about 3 mm from the tip.

**Conclusions:**

A knot formation of an epidural catheter placed at the thoracic level was experienced. Limiting the length of catheter placement may prevent knot formation.

## Background

The knot formation in the epidural catheter is a rare complication that can result in difficult removal. Although most epidural catheter knot formation has been reported in lumbar epidural catheter placement used for obstetric analgesia and/or anesthesia [1], knot formation in a thoracic epidural catheter for a nonobstetric procedure has been experienced.

## Case presentation

A 72-year-old woman was admitted to the hospital for gallbladder polyps and was scheduled for laparoscopic cholecystectomy. A possibility of undergoing laparotomy due to intra-abdominal adhesions exists because the patient had a history of sigmoidectomy and transverse colon resection for colorectal cancer. Therefore, the patient was planned to be operated on under general anesthesia combined with epidural anesthesia for postoperative analgesia.

The patient was placed in the left lateral position and an epidural puncture with a 17-gauge Tuohy needle (B. Braun Melsungen A.G., Melsungen, Germany) was performed using a paramedian approach from the Th10–Th11 intervertebral space. The loss of resistance technique with normal saline was used for the identification of the epidural space. The epidural space was identified at a 4-cm depth, and a 19-gauge FX catheter (B. Braun Melsungen A.G.) was advanced to 8 cm from the needle tip. There was no resistance when inserting the epidural catheter. The Tuohy needle was then removed, and the epidural catheter was withdrawn 1 cm and placed 7 cm into the epidural space.

General anesthesia was induced and maintained with propofol, desflurane, remifentanil, and rocuronium after epidural catheter placement. The patient did not need to undergo laparotomy because no adhesions exist around the gallbladder, and the operation was completed without any particular event. A total of 8 mL of 0.5% levobupivacaine was intraoperatively bolus administered through the epidural catheter, followed by continuous administration of 200, 20, and 80 mL of 0.25% levobupivacaine, fentanyl, and normal saline, respectively. After surgery, pentazocine (7.5 mg, intravenous drip) was used once and acetaminophen (500 mg, oral) was used twice, but the pain was generally well-controlled.

The surgeon tried to remove the epidural catheter 2 days after the surgery. However, it was not removed due to resistance, and the anesthesiologist was called. The catheter gradually came out although resistance was still noted when the anesthesiologist carefully pulled it while strongly bending the patient’s body to the right. Consequently, the catheter was finally removed without any problems except for some discomfort at the insertion site during removal, and no neurological abnormality occurred.

**Fig. 1:**
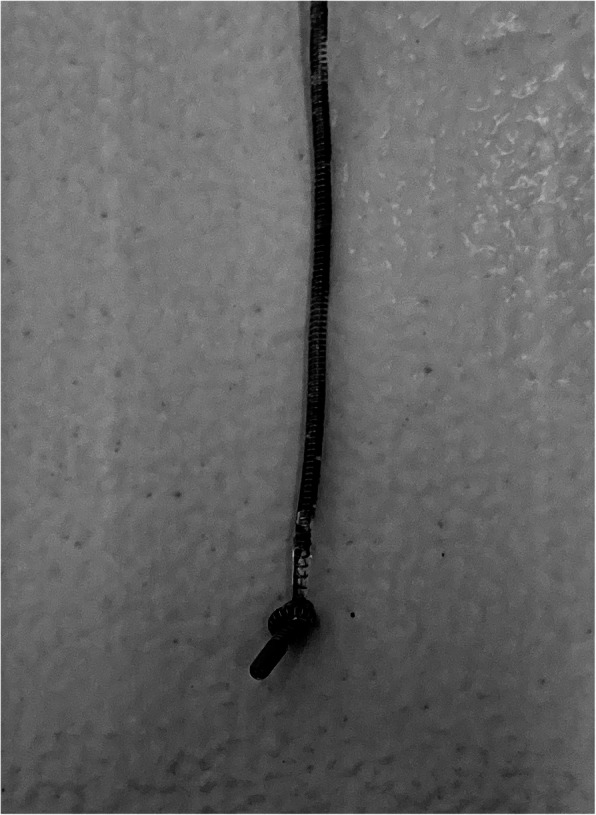
Removed epidural catheter. A knot was formed at about 3 mm from the tip

**Fig. 2:**
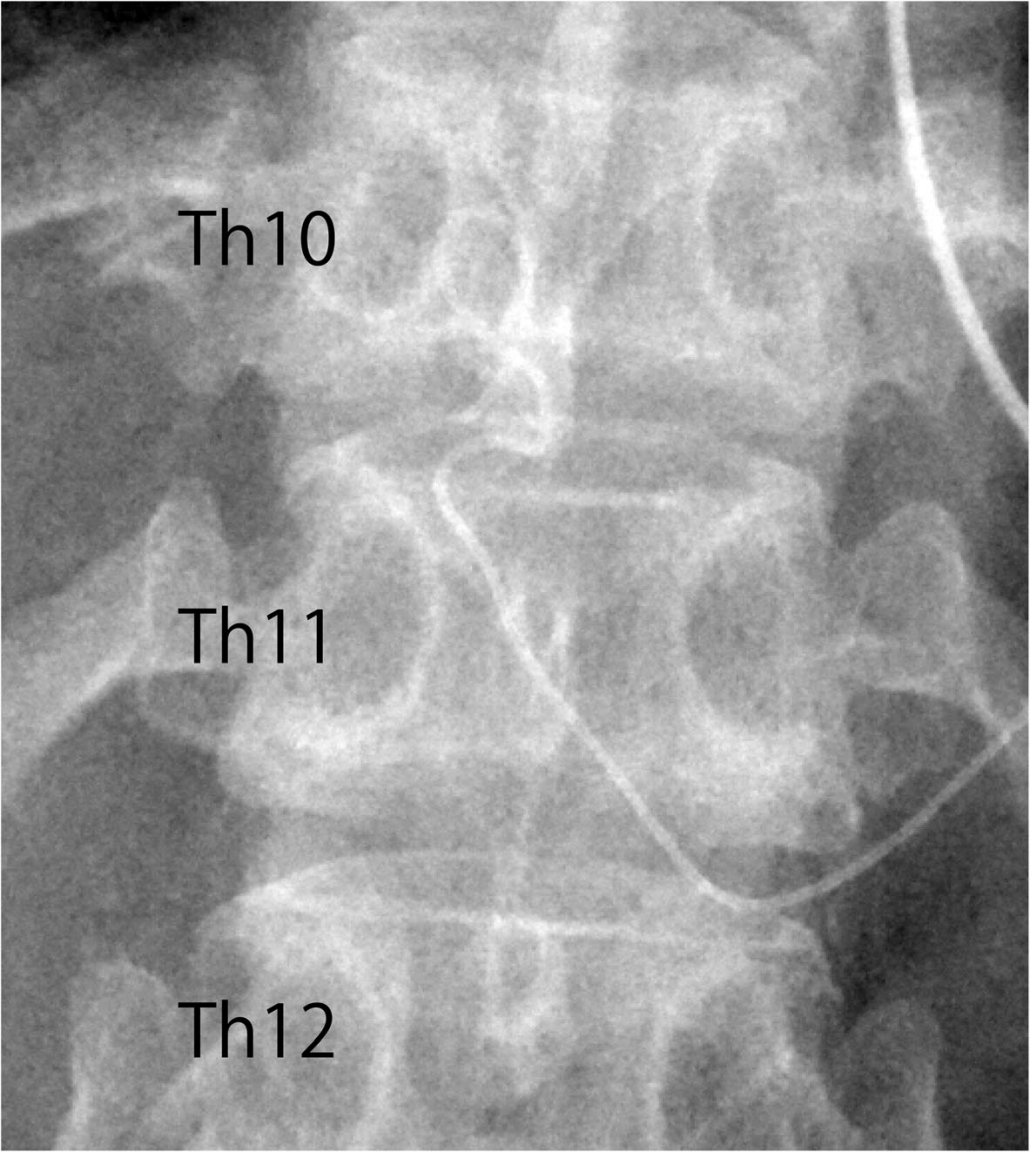
A plain abdominal X-ray taken after the surgery

Observation of the removed catheter showed that a hard single knot was formed at about 3 mm from the tip (Fig. 1). A plain abdominal X-ray taken after the surgery showed that the indwelling catheter was running in a loop (Fig. 2).

## Discussion

Epidural catheter knot formation is a very rare complication of indwelling epidural catheters and has an estimated incidence of 0.0015% [2]. In a case report and literature review by Brichant et al. [1], 18 cases of epidural catheter knot formation were identified. The epidural catheter was placed at the lumbar level in most cases, and an epidural catheter placed at the thoracic level formed a knot in only one case. To our knowledge, this is the second case report of knot formation in an epidural catheter placed at the thoracic level.

The reason why knot formation is less frequent with thoracic epidural catheters is not clear; however, one possible reason is that the catheter advances differently in the epidural space in the thoracic and lumbar regions. Muneyuki et al. [3] reported that an indwelling thoracic epidural catheter was less likely to curl, bend, or kink in epidural space than an indwelling lumbar epidural catheter, and a greater amount of the catheter can be inserted without coiling. They suggested that this difference in catheter travel is caused by the difference in the angle of insertion of the needle. In the lumbar region, the epidural needle impinges on the dura at a right angle, whereas in the thoracic region, the needle is inserted at an obtuse angle to the epidural canal, which may make it easier for the catheter to be inserted straight.

The catheter in the epidural space in the current case was observed to be coiling on postoperative abdominal plain radiography, which may have led to knot formation when the catheter was attempted to be removed. Several authors recommend that the length of catheter placement in the epidural space should be limited to minimize the risk of complications (e.g., catheter dislodgement, intravenous cannulation, or knot formation) [4–6]. To prevent catheter loop formation, indwelling lumbar epidural catheters should not be placed into the epidural space beyond 5 cm [5, 7]. As for the thoracic epidural, it has been reported that catheters tend to insert straighter compared to inserting into the lumbar region, and inserting up to 10 cm without forming a loop is possible [3]. However, a more recent study reported that the thoracic epidural catheter forms a loop at 4.9–7.4 cm, depending on the angle of approach [8]. In fact, in the present case, a loop was formed after 7 cm of catheter placement, and knot formation occurred during removal. This case suggests that loop and knot formations occur even when the length of the indwelling thoracic epidural catheter is less than 10 cm.

Pulling gently with a constant force to prevent catheter breakage is necessary when removing an epidural catheter. Although catheters were successfully removed in most of the reported cases of knot formation, applying traction on the catheter resulted in catheter breakage in about 30% of cases [1]. In our case, the catheter came out gradually by pulling gently, but if excessive force is required to remove the epidural catheter and the catheter is stretched, visualization of the catheter by plain X-ray or computed tomography should be considered to check for knot formation.

In conclusion, a knot formation of an epidural catheter placed at the thoracic level was experienced. Limiting the length of catheter placement may prevent knot formation. Moreover, visualization of the catheter by plain X-ray or computed tomography should be considered if the epidural catheter is difficult to remove.

## Data Availability

Not applicable.
